# X chromosome-wide analysis identifies DNA methylation sites influenced by cigarette smoking

**DOI:** 10.1186/s13148-016-0189-2

**Published:** 2016-02-24

**Authors:** Daniella Klebaner, Yunfeng Huang, Qin Hui, Jacquelyn Y. Taylor, Jack Goldberg, Viola Vaccarino, Yan V. Sun

**Affiliations:** Department of Epidemiology, Emory University Rollins School of Public Health, 1518 Clifton Road NE #3049, Atlanta, GA 30322 USA; School of Nursing, Yale University, Orange, CT USA; Vietnam Era Twin Registry and Department of Epidemiology, University of Washington School of Public Health, Seattle, WA USA; Department of Biomedical Informatics, Emory University School of Medicine, Atlanta, GA USA

**Keywords:** X chromosome, Epigenome, Epigenetics, DNA methylation, Methylome, Smoking, Epigenome-wide association study, Twin, Heritability

## Abstract

**Background:**

Tobacco smoking is a major cause of chronic disease worldwide. Smoking may induce cellular and molecular changes including epigenetic modification, with both short-term and long-term modification patterns that may contribute to phenotypic expression of diseases. Recent epigenome-wide association studies (EWAS) have identified dozens of smoking-related DNA methylation (DNAm) sites. However, the X chromosomal DNAm sites have been largely overlooked due to a lack of an analytical framework for dealing with the sex-dimorphic distribution. To identify novel smoking-related DNAm sites on the X chromosome, we examined the modality of each X chromosomal DNAm site and conducted a sex-specific association study of cigarette smoking.

**Results:**

We used a discovery sample of 139 middle-age twins, and three replication samples of 78 twins, 464 and 333 unrelated individuals including 47, 17, 22, and 89 current smokers, respectively. After correction for multiple testing, the top smoking-related DNAm sites in *BCOR* and *TSC22D3* were significantly hypermethylated and hypomethylated, respectively, among current smokers. These smoking-associated sites were replicated with meta-analysis *p*-values of 9.17 × 10^−12^ and 1.61 × 10^−9^. For both sites, the smoking effects on methylation levels were larger in males than that in females.

**Conclusions:**

Our findings highlight the importance of investigating X chromosome methylation patterns and their associations with environmental exposures and disease phenotypes and demonstrate a robust statistical methodology for such study. Existing EWAS of human diseases should incorporate the X chromosomal sites to complete a comprehensive epigenome-wide scan.

**Electronic supplementary material:**

The online version of this article (doi:10.1186/s13148-016-0189-2) contains supplementary material, which is available to authorized users.

## Background

Cigarette smoking is an established risk factor for morbidity and mortality; up to one third of adults worldwide are exposed to tobacco use, with smoking-related deaths still on the rise [1–3]. Despite successful public health efforts to reduce smoking prevalence in the USA, 17.8 % of the US adult population smoked as of 2013 [4]. Cigarette smoking negatively impacts health through numerous biological mechanisms; while many of these pathways have been studied and enumerated at length, smoking-mediated epigenetic changes and subsequent health effects have not been fully explored.

Epigenetic modifications, through DNA methylation (DNAm) and other molecular mechanisms, can regulate gene expression levels and represent an important molecular mechanism underlying disease development. Environmental factors, along with genetics and stochastic processes, are the primary sources of epigenetic variation [5, 6]. Specifically, epigenetic mechanisms may mediate environmental risks such as smoking [7–10], pollutants [11–13], and lifestyle behaviors [14–16] for chronic disease development.

DNA methylation, a subtype of epigenetic modification, refers to the addition of a methyl group to cytosine nucleotides [17, 18]. Numerous epigenome-wide association studies (EWAS) have demonstrated a relationship between smoking and autosomal DNA methylation at certain CpG sites in adults [8–10, 19–25]; certain sites, such as those on the *F2RL3* [7, 9] and *AHRR* [8–10] genes, have been identified as markers of smoking and cardiovascular disease phenotypes [7].

However, the X chromosome is often excluded from such epigenome-wide approaches due to hemimethylation in females as a product of X chromosome inactivation (XCI) [26], despite the fact that the X chromosome harbors hundreds of protein-coding genes heavily involved in biological processes and gene-specific DNA methylation [27, 28]. As the eighth largest chromosome of human genome, the X chromosome accounts for 5 % of human genome. However, almost 7 % of diseases with a Mendelian pattern of inheritance (322 out of 4754) have been linked to the X chromosome according to Online Mendelian Inheritance in Man (OMIM) [29]. Genome-wide association studies (GWAS) have identified numerous X chromosomal SNPs associated with human traits and diseases such as male-pattern baldness [30], Graves’ disease [31], rheumatoid arthritis [32], prostate cancer [33], type 2 diabetes [34], and red blood cell traits [35]. Additionally, numerous human diseases have been linked to aberrant epigenetic modification related to XCI [28], and X-linked genes may affect the development of disease via the epigenetic regulation of specific genes [36, 37].

As a hallmark of XCI, hemimethylation of the X chromosome results in a bimodal distribution strongly associated with sex [38, 39]. As a result, findings of X chromosome associations have been limited by a lack of analytical methods to account for XCI [6, 40].

From methylome-wide data of human tissues, a large number of loci on the X chromosome showed sex-specific dimorphism of DNA methylation [38, 39, 41]. Thus, any study of X chromosome-wide DNA methylation needs to clearly account for sex dimorphism in its statistical methods.

By combining sex stratification and statistical testing for unimodality of methylation levels, we sought to address this gap in the literature by characterizing smoking-related DNA methylation on the X chromosome. We aimed to precisely estimate the association between cigarette smoking and DNA methylation on the X chromosome in a well-characterized population and to replicate the association to demonstrate the generalizability of our approach. Our analytic methods may serve as a starting point for future analyses of epigenetic modification on the X chromosome.

## Results

The average age of participants was 55.7 in the discovery twin cohort, 55.4 in the second twin cohort, 54.6 in the GEO GSE50660 dataset, and 52.8 in the GEO GSE42861 dataset. Out of the 139 and 78 twins, 34 and 22 % were current smokers, respectively. Among the GEO GSE50660 and GSE42861 cohorts, 4.7 and 26.7 % were current smokers, respectively. The sex-specific statistics of age, smoking status, and body mass index (BMI) are summarized in Table 1.

**Table 1:** Demographic information for the discovery and replication cohorts stratified by sex

Variable	*N* (%) or mean (SD)					
	Twins I (*n* = 139)	Twins II (*n* = 78)	GSE50660 male (*n* = 327)	GSE50660 female (*n* = 137)	GSE42861 male (*n* = 95)	GSE42861 female (*n* = 238)
Current smoker						
Yes	47 (34 %)	17 (22 %)	15 (4.6 %)	7 (5.1 %)	23 (24 %)	66 (28 %)
No	92 (66 %)	61 (78 %)	312 (95 %)	130 (95 %)	72 (76 %)	172 (72 %)
Age	55.7 (3.3)	55.4 (3.3)	54.6 (6.8)	57.3 (5.9)	55.5 (10.5)	51.7 (11.7)
BMI	28.9 (4.3)	29.7 (4.1)	NA	NA	NA	NA

*NA* not available

The histograms of the overall *β*-value distribution of X chromosomal sites in males and females (Additional file 1: Figure S1) are consistent with bimodal distribution due to XCI: males show two peaks at <0.1 and >0.8 while females show a large peak of mean *β*-value close to 0.5. Among the 139 male twins, we used the *dip* statistic to assess the unimodality of all X chromosomal DNAm sites. After excluding sites overlapping with SNPs, we identified 14 DNAm sites multimodally distributed among males after multiple testing correction (i.e., Bonferroni-corrected empirical *p* value <0.05). Using a much less stringent threshold to reject unimodality (unadjusted empirical *p* value <0.05), only 47 sites (less than 0.5 %) were found to be potentially multimodal. In contrast, in the sample with both males and females, more than 35 % of DNAm sites on the X chromosome presented multimodal distribution using stringent Bonferroni correction (unpublished).

An X chromosome-wide epigenetic association study of current smoking compared to non-current smoking (i.e., past plus never smokers) in the discovery cohort identified two significant sites, cg07764473 in the gene *BCOR* and cg21380860 in the gene *TSC22D3*, using a false discovery rate (FDR) threshold of 5 % to control for multiple comparisons. Although the scales of effects are different between the *M*-value and *β*-value analyses, the levels of significance were highly consistent between the two quantitative measurements of DNA methylation (Table 2). Though all analyses were performed using both *β*- and *M*-values, we chose to focus on the results of *β*-value analyses in the following sections since they have a direct biological interpretation. Quantile-quantile plots comparing the observed to the expected *p*-values for this analysis showed moderate inflation (genomic control inflation factor = 1.1), with the two significant sites visibly apparent above the curve (Additional file 1: Figure S1). The Manhattan plot for the X chromosome-wide analysis comparing current smokers to non-current smokers in the twins discovery cohort depicted the notable significance level of the two selected CpG sites at the FDR-adjusted threshold of 0.05 (Additional file 1: Figure S2). Corrected for multiple testing, we did not identify any significant DNAm sites on X chromosome associated with pack-years among smokers. Adjusted for age, BMI, and cell type proportions, cg07764473 (*BCOR*) was not significantly associated with pack-years at alpha level of 0.05, and cg21380860 was marginally significant (*p*-value of 0.02). Neither site was statistically significant when current smoking status was adjusted in the model.

**Table 2:** Smoking-related DNAm sites on X chromosome in males

CpG sites	Genes	Datasets	*β*-value				*M*-value			
			Beta	SE	T-stat	*p*-value	Beta	SE	T-stat	*p*-value
cg07764473	*BCOR*	Twins I	0.058	0.009	6.02	1.21 × 10^−7^	0.35	0.058	6.05	1.05 × 10^−7^
		Twins II	0.030	0.013	2.42	0.02	0.18	0.078	2.25	0.03
		GSE50660	0.028	0.016	1.78	0.07	0.16	0.091	1.80	0.07
		GSE42861	0.037	0.014	2.57	0.01	0.22	0.084	2.58	0.01
		Meta	0.043	0.006	6.82	9.17 × 10^−12^	0.25	0.037	6.83	8.57 × 10^−12^
cg21380860	*TSC22D3*	Twins I	−0.020	0.004	−5.14	3.30 × 10^−6^	−0.19	0.037	−5.04	4.71 × 10^−6^
		Twins II	−0.014	0.007	−2.14	0.04	−0.13	0.059	−2.13	0.04
		GSE50660	−0.015	0.008	−1.91	0.06	−0.11	0.061	−1.86	0.06
		GSE42861	−0.014	0.008	−1.62	0.11	−0.10	0.065	−1.55	0.12
		Meta	−0.017	0.003	−6.03	1.61 × 10^−9^	−0.15	0.026	−5.81	6.25 × 10^−9^

**Fig. 1:**
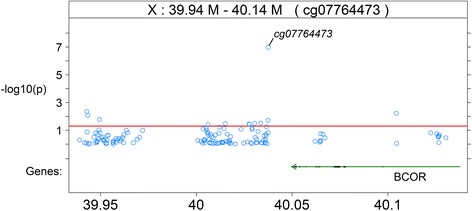
Regional plot of DNAm site cg07764473 of *BCOR* gene in male twins. The *red horizontal line* indicates the threshold of *p* value 0.05

Within the *BCOR* region, there were multiple DNAm sites with *p*-value less than 0.05, particularly downstream (3′-UTR) of the genic region (Fig. 1). Within a 200-kb region around cg07764473, 12 DNAm sites had *p*-value less than 0.05 out of 139 sites (i.e., 9 %). Within the smaller block immediately neighboring cg07764473, 8 out of 74 DNAm sites (11 %) had a *p*-value less than 0.05. Within the flanking region immediately neighboring cg21380860 (*TSC22D3*), 4 out of 51 DNAm sites (7.8 %) had a *p* value less than 0.05. DNAm sites cg07764473 and cg21380860 presented a unimodal distribution in males indicated by their non-significant empirical *p*-values (0.92 and 0.61, respectively) from the *dip* test.

**Fig. 2:**
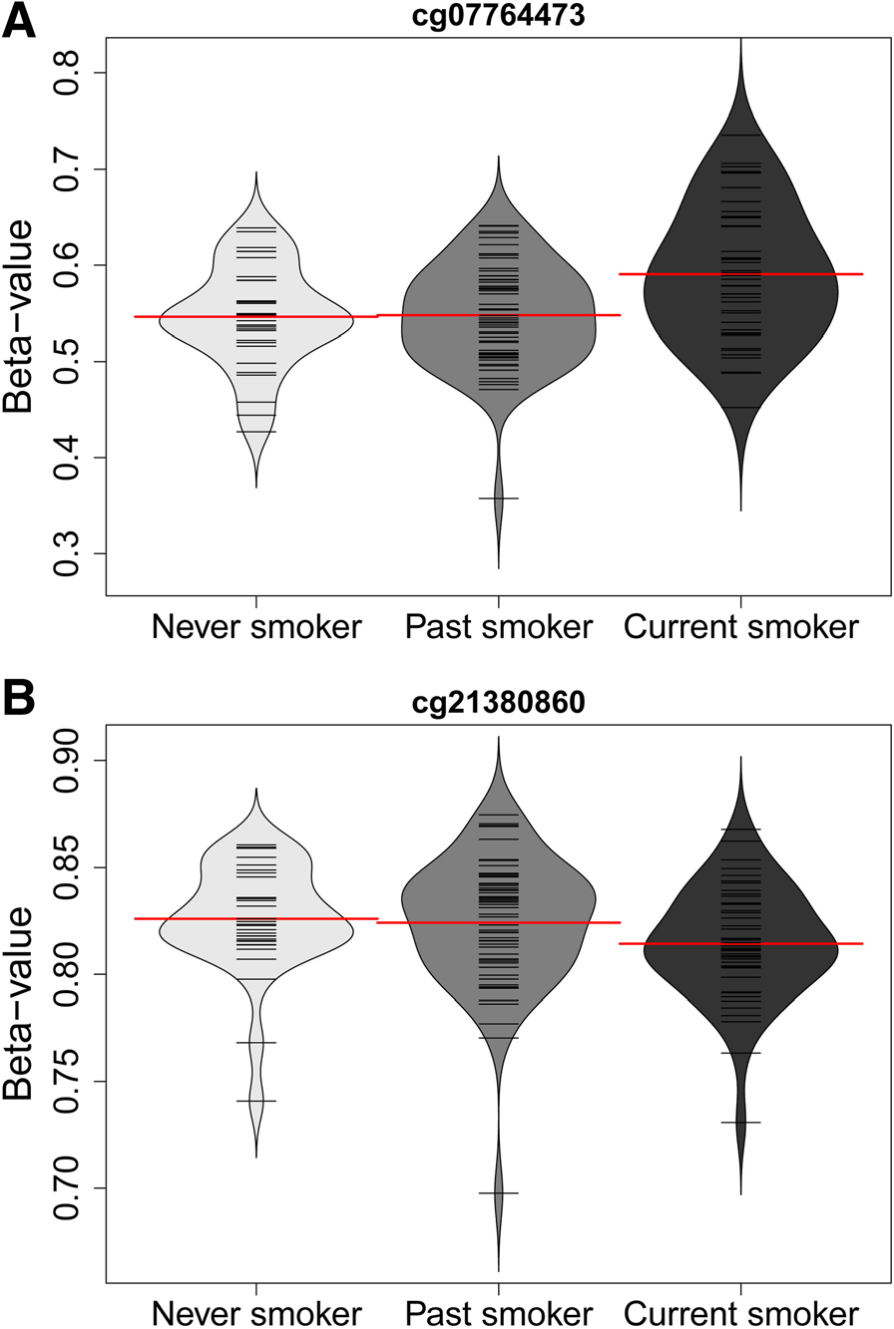
Smoking-related DNA methylation levels of **a** cg07764473 (*BCOR*) and **b** cg21380860 (*TSC22D3*) among never (*light gray*), past (*medium gray*), and current smokers (*dark gray*) in male twins. Each *black line* indicates an individual’s DNA methylation level measured by *β*-value. *Red line* indicates the mean level of *β*-values of each group of smokers

The bean plots of the *β*-values in the discovery cohort showed an apparent increasing difference between never, past, and current smokers corroborating the significant effect of smoking on DNA methylation (Fig. 2). Current smoking increased the DNA methylation in site cg07764473 and decreased DNA methylation in site cg21380860. For both sites, the difference of DNA methylation between never and past smokers is undistinguishable.

To understand the genetic and environmental contributions to these two smoking-related DNAm sites, we used a structural equation modeling (SEM) method implemented in OpenMX [42] to partition the additive genetic, common environmental, and unique environmental variance in a total of 81 monozygotic (MZ) and 27 dizygotic (DZ) twin pairs. Both sites, cg07764473 and cg21380860, showed substantial contributions from common (*c*^2^ = 38 % and 44 %, respectively) and unique environmental factors (*e*^2^ = 62 % and 56 %, respectively), without any contribution from the additive genetic factors (*a*^2^ = 0). By testing the differential methylation between 23 MZ twin pairs discordant for current smoking status, we confirmed that the unique environmental factors most likely drive the identified epigenetic association with cigarette smoking. DNAm site cg07764473 is hypermethylated among current smokers with mean *β*-value difference of 0.054 (*p*-value of 8.29 × 10^−4^). DNAm site cg21380860 is hypomethylated among current smokers with mean *β*-value difference of 0.0083 (*p*-value of 0.15).

**Fig. 3:**
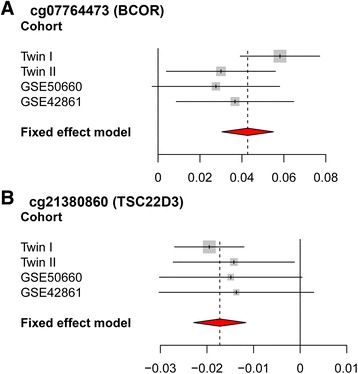
Forest plots of the smoking-related DNAm sites in males from the discovery and three replication samples using *β*-value. **a** cg07764473 (*BCOR*). **b** cg21380860 (*TSC22D3*)

To replicate the two significant associations with current smoking in the male twins, we examined the same DNAm sites, cg07764473 and cg21380860, from the three samples described (Table 2). For both DNAm sites, the associations in all three replication samples had the same direction of effects as the discovery sample (Table 2 and Fig. 3). The associations with current smoking were statistically significant (*p*-value <0.05) except in the male sample of GSE50660 (*p*-value <0.1), which had the least number of current smokers and therefore limited power (*N* = 15). The analyses using *β*-values and *M*-values showed consistent significance levels across all samples for both DNAm sites (Table 2).

We combined the results of *β*-value analyses from the discovery and replication samples using a fixed effect meta-analysis approach (Fig. 3). For DNAm site cg07764473, the combined effect was a 0.043 increase in the *β*-value with a standard error (se) of 0.006 (*p*-value of 9.17 × 10^−12^). For DNAm site cg21380860, the combined effect was a 0.017 mean decrease in the *β*-value with a se of 0.03 (*p*-value of 1.61 × 10^−9^). We did not observe significant heterogeneity across the summary statistics of these four samples. The *I*^*2*^ of cg07764473 and cg21380860 were 34.7 % (*p*-value of 0.204) and 0 % (*p*-value of 0.843), respectively.

**Fig. 4:**
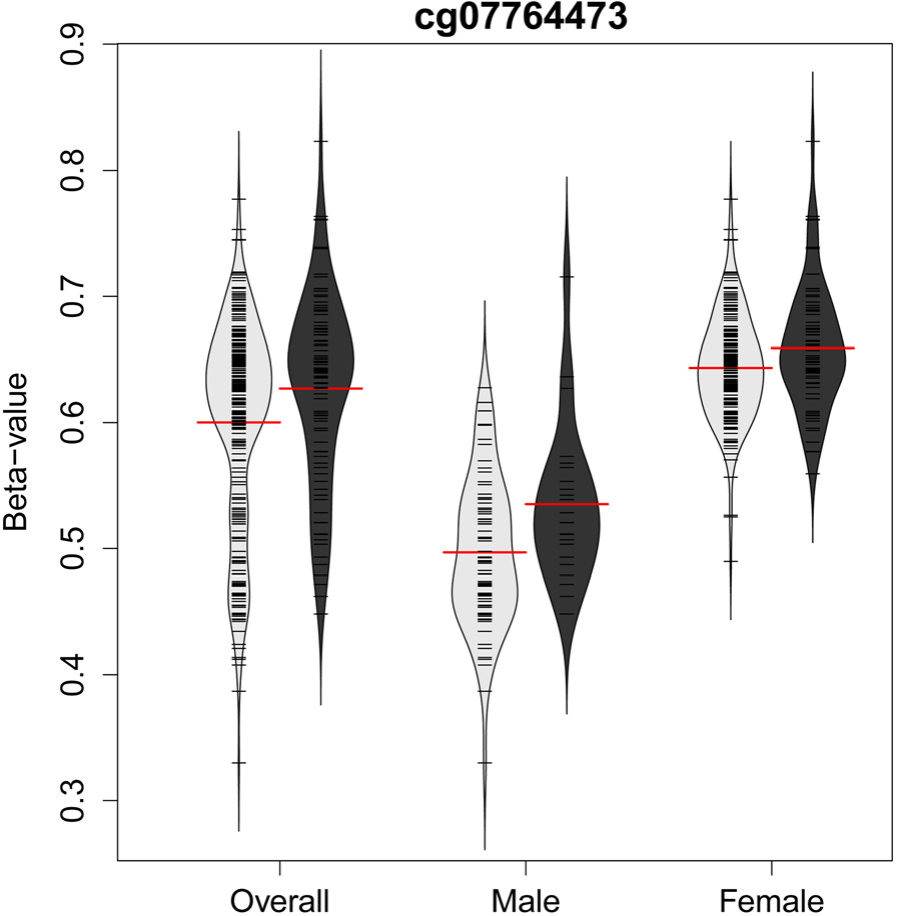
Sex stratified and pooled methylation levels of cg07764473 (*BCOR*) in current (*dark gray*) and non-current smokers (*light gray*). Each *black line* indicates an individual’s *β*-value of cg07764473. *Red line* indicates the mean level of *β*-values of each group of smokers

In addition to the replication of two smoking-related DNAm sites in males, we examined the epigenetic associations with current smoking status in 238 females (66 current smokers) from dataset GSE42861. Adjusted for age, cg07764473 in the *BCOR* gene was significantly associated with current smoking using both *β*-values and *M*-values with *p*-values of 0.013 and 0.010, respectively. Compared to the smoking association in 95 males from the same dataset (Table 2), the DNAm site was hypermethylated in both males and females. However, the effect size in the females (*β*-coefficient of 0.017) was about half of that seen in the male cohort (*β*-coefficient of 0.037) using the *β*-values. The results were similar (0.11 and 0.22 in females and males, respectively) from the analysis of *M*-values. In addition, the methylation level of cg07764473 in females was higher (mean of *β*-value is 0.65) than that found in males (mean of *β*-value is 0.51) with a *p*-value less than 2.2 × 10^−16^. In fact, the variation between sexes was much larger than the variation between smoker categories (Fig. 4). For DNAm site cg21380860 in gene *TSC22D3*, the directionality of effect was consistent between males and females (i.e., hypomethylated in both sexes). Although the effect sizes of cg21380860 associations were also smaller in females than that in males, the associations were not statistically significant (i.e., *p*-value >0.05). Contrary to what we found for the cg07764473 site, DNAm site cg21380860 showed a higher level of methylation in males (mean *β*-value was 0.76) than that in females (mean *β*-value was 0.71).

## Discussion

After careful examination of the modality of X chromosomal data, we performed sex-stratified epigenetic association analysis of cigarette smoking. Two sites were significantly associated with current smoking in the discovery cohort and were successfully replicated in three independent samples. DNAm site cg07764473 (X: 40,037,510 bp) is located in the BCL6 co-repressor (*BCOR*) gene. It was consistently hypermethylated (i.e., higher level of methylation) in current smokers. *BCOR* is expressed ubiquitously throughout human tissues and is best known for causing X-linked oculofaciocardiodental syndrome when mutated [43]. *BCOR* plays a significant role in gene expression in conjunction with a complex of histone modification proteins which epigenetically modify chromatin [44, 45]. Our results show that cigarette smoking results in hypermethylation of *BCOR*, which may be responsible for decreased expression of *BCOR* induced by exposure to smoke extract [46]. Given *BCOR*’s role in regulating gene expression and gene silencing, it is highly plausible that cigarette smoking may lead to ubiquitously altered gene expression and disease phenotypes [44]. *BCOR* mutations have been associated with diseases such as acute myeloid leukemia, and decreased expression of *BCOR* may similarly lead to poor health outcomes resulting from a lack of gene expression regulation [45]. Furthermore, *BCOR*’s major role in embryonic stem cell differentiation may be significantly altered by inheritance of downregulated expression as a result of maternal smoking [43].

DNAm site cg21380860 (X: 106,958,499 bp) is located in the glucocorticoid-induced leucine zipper protein (*TSC22D3*) gene. It was hypomethylated among current smokers across all samples, though the association was insignificant using the discordant MZ twin approach; this insignificant result may be due to a small sample size of discordant MZ twins (*N* = 23). *TSC22D3* has been proposed as a regulator of immunity, adipogenesis, and renal sodium handling [47]. Mice with deficient *TSC22D3* genes were shown to be infertile, and in humans, the gene is a known tumor suppressor which is often silenced in cancers via hypermethylation [47–49], though only hypomethylation was observed in our samples of peripheral leukocytes. However, patterns of DNA methylation can be different across tissue types and gene regions.

The smoking effect on cg21380860 (*BCOR*) was doubled in males compared to females, with a lower level of methylation in males. Since females carry two X chromosomes, with one inactivated by XCI, smoking-related hypermethylation can only affect one chromosome, with less methylation on the active copy. The DNAm site on the inactive copy cannot be further methylated due to a saturated methylation level, or inaccessibility to methylation enzymes and cofactors. As a result, the observed average effect on two chromosomes is likely diluted by the inactive copy. In association studies of X chromosome sites, the statistical power of detecting epigenetic effects favors males over females with comparable sample sizes.

Epigenomic profiles are cell type-specific [38, 50]. Although several subtypes of peripheral blood leukocytes (PBLs) may respond to cigarette smoking differently at the epigenetic level, we were not able to investigate the epigenomic profile of X chromosome in all of the subtypes. Instead of profiling each cell subtype, we investigated the PBLs as an aggregated summary of multiple cell types while adjusting for the cell type proportions as a potential confounder [51]. However, residual confounding may not be fully accounted in the specific subtype proportion estimates. The recent reference free adjustment method [52] may improve the estimation of unmeasured PBL subtypes but the application in related individuals (e.g., twins) need to be further evaluated.

Significant findings in epigenetic association studies should be validated in replicate samples as recommended for genetic association studies [53] in order to minimize false positive results [54] or analytical bias [55]. Our X chromosome findings were replicated in both men and women. However, the sample size of the discovery cohort limited our ability to identify smaller smoking effects. Additionally, sex-specific DNAm changes on the X chromosome suggest a smoking-sex interaction effect, but we were not able to formally examine the X chromosome-wide interaction effects due to the limited number of female subjects with both DNAm and phenotypic data. Future studies on large populations with both male and female smokers are needed to fully understand the effect of smoking on the epigenetic profile of X chromosome. For the success of X chromosome-wide epigenetic association study, we encourage the sharing of raw DNAm data with all X chromosomal sites.

## Conclusions

Our findings highlight the need for further investigation of X chromosome methylation patterns and their associations with environmental exposures and disease phenotypes. Discovery of pertinent CpG sites on the X chromosome presents an opportunity to understand health outcomes and their presently unstudied mediation through gene-environment interactions. Recent EWAS combined thousands of samples from multiple cohorts [56–58], which should be well-powered to identify X chromosomal DNAm sites associated with disease traits [59]. As such, studying methylation on the X chromosome with robust statistical methods will allow discovery of novel epigenetic mechanisms affecting disease phenotypes, particularly for sex-biased traits.

## Methods

### Study population

*Emory Twin Study*: For our primary cohort, we used DNA methylation and phenotype data from the Emory Twin Study (ETS). The ETS consists of 307 middle-aged male Caucasian MZ and DZ twin pairs from the Vietnam Era Twin Registry [60] who were born between 1946 and 1956 [61, 62]. All twins were examined in pairs at the Emory University General Clinical Research Center between 2002 and 2010. Twins were given the same diet the night before the assessments and instructed to refrain from smoking. All measurements were performed in the morning after an overnight fast, and both twin pairs were tested at the same time. All medications were held for approximately 24 h prior to testing. Biochemical assays for each twin pair were processed in the same analytical run. A medical history and a physical exam were obtained from all twins. Weight and height were measured and used to calculate BMI. Cigarette smoking was classified into current smoker (any number of cigarettes) versus never or past smoker. Venous blood samples were drawn for the collection of plasma and PBL and stored at −80 °C until the biomedical assay. Information on zygosity was determined by DNA analysis. Genomic DNA samples were successfully epityped using the Illumina HumanMethylation450 Beadchip (450K) in two batches of 142 and 78 twins, respectively. The ETS was approved by the Emory Institutional Review Board, and all participants signed an informed consent.

*Datasets from Gene Expression Omnibus* (*GEO*): To replicate our primary analyses, we downloaded a GEO dataset (GSE50660) containing information on cigarette smoking [63], DNA methylation, and phenotype information on 464 individuals, 327 of whom were male. We also downloaded and analyzed another GEO dataset GSE42861, which measured the DNA methylome of peripheral blood using the same Illumina 450K chip [64], and included DNAm profiles of rheumatoid arthritis (RA) cases and controls. We performed analyses using data on smoking status and X chromosome DNAm only from the 333 controls. The genomic DNA samples from both studies were similarly epityped using the Illumina 450K methylation chip following the manufacturer’s instructions. Raw data were normalized using Illumina’s control probe scaling procedure and converted to methylation *β*-values. Detection *p*-values were calculated to identify and exclude failed probes as per Illumina’s recommendations [64].

### DNA methylation methods

The EZ DNA Methylation Kit (Zymo Research, Orange CA) was used to bisulfite-convert 0.5 μg of genomic DNA per sample from peripheral blood leukocytes (PBLs). Bisulfite-converted DNA samples were whole-genome amplified, enzymatically fragmented, and purified. Samples were then hybridized in batches of 12 to the BeadChip, which contains locus-specific DNA oligomers. The arrays were fluorescently stained, scanned, and assessed for fluorescence intensities at each bead site. Each DNAm site was quantified using beta (*β*)-values:

$$ \beta\ \hbox{-} \mathrm{value}=\frac{ \max \left({I}_{i\kern0.5em \mathrm{methylated}},0\right)}{\left|{I}_{i\kern0.5em \mathrm{methylated}}\right|+\left|{I}_{i\kern0.5em \mathrm{unmethylated}}\right|+\alpha } $$

The *β*-values generated by GenomeStudio software were used for data pre-processing and quality control. These values were continuous variables ranging from 0 to 1, which represent the ratio of fluorescence intensity of the methylated and unmethylated sites. Using the detection *p*-value threshold of 0.001, two individual samples with a missing rate above 5 % were excluded, resulting in 140 and 78 eligible twins in the following analyses. No sample was detected with control probe values greater than 4 standard deviations from its mean value. CpG sites were excluded from analyses if they had missing rate above 5 % (*N* = 119), overlapped with single nucleotide polymorphisms (*N* = 370) base on Illumina’s 450K annotation, or were not uniquely mapped to the reference genome (*N* = 774) [65].

We transformed DNA methylation *β*-values into *M*-values by performing a logit transformation, based on evidence showing the improved performance of *M*-values in the detection rate and true positive rate for both unmethylated and methylated CpG sites [66]:

$$ M\hbox{-} \mathrm{value}={ \log}_2\left(\frac{\beta }{1-\beta}\right) $$

The *M*-value is a commonly used measurement in microarray analysis that was more recently adapted for use in DNA methylation array data due to its approximately homoscedastic distribution, making it a more statistically valid estimator [66, 67].

### Assessment of multimodality

XCI and subsequent hemimethylation of the X chromosome sites is presumed to result in a bimodal distribution strongly associated with sex. Since males have only one copy of X chromosome, a DNAm site is either unmethylated (*β*-value close to 0) or methylated (*β*-value close to 1); in contrast, a large number of DNAm sites on X chromosome are hemimethylated (*β*-value close to 0.5) in females due to XCI (one copy of X chromosome is unmethylated and the other copy is methylated). We plotted a histogram of *β*-values for males and females to visually compare the distribution of methylation levels between males and females. We used the *Hartigan*’*s dip* statistic to test for multimodality among a sample of only males, as well as a sample including both males and females, to assess the extent of sex-associated bimodality. Multimodality in the sample with both sexes compared with unimodality in the sample of males would indicate XCI-induced hemimethylation among females and sex-related multimodality.

The *dip* test measures multimodality in a sample by the maximum difference, over all sample points, between the empirical distribution function and the unimodal distribution function that minimizes that maximum difference. The *dip* statistic is defined as $\mathrm{dip}=\underset{F\in U}{ \inf}\underset{x}{ \sup}\left|F(x)-{F}_n(x)\right|$, the maximum difference between the empirical distribution function *F*_*n*_, and the closest distribution *F* from the class of all unimodal distributions *U* [68, 69]. A large *dip* indicates multimodality. The uniform distribution is the asymptotically least favorable unimodal distribution, and the distribution of the test statistic is determined asymptotically and empirically when sampling from the uniform distribution [68].

The details of the *dip* statistic and the proof were originally described by Hartigan and Hartigan [68]. The *dip* statistic of the *β*-values of each DNAm site was calculated using the R package “diptest.” The *p*-value of the *dip* statistic can be calculated by comparing to the distribution generated from the random uniform distribution. An accurate empirical *p*-value of a given *dip* statistic has to be computed by generating a large number of simulations of the same sample size [68]. We calculated the distribution of *dip* statistic from 10^8^ uniform distributed samples. The empirical *p*-value is determined by comparing the observed *dip* statistic to 10^8^ dip statistics. The empirical *p*-value is set to be <10^−8^ when the observed *dip* statistic is larger than the largest *dip* statistic from 10^8^ simulated uniform distributions.

The null hypothesis *H*_0_ is that the observed distribution is unimodal. The null hypothesis of unimodality is rejected when the empirical *p*-value is less than a significance threshold. Because a large number of DNAm sites are tested, we applied a Bonferroni-adjusted *p*-value of 0.05 to restrict false positive findings. This approach was previously applied to exclude DNAm sites with multimodal distributions from EWAS [9, 70, 71].

### Epigenetic association analysis

To identify smoking-related DNAm sites in the two twin cohorts, we used linear mixed models with the *β*-value/*M*-value as the dependent variable and smoking status as the primary independent variable. Pack-years were calculated by multiplying the number of years smoked and the average pack (number of cigarettes divided by 20) of cigarettes smoked per day. We included random effects to account for the chip/batch effect, as well as the co-twin relationship. All associations were adjusted for age in years and BMI.

We estimated the proportions of PBL subtypes for each twin sample using an algorithm developed by Houseman et al. [51]. The proportions of six different cell types, including granulocyte, monocyte, natural killer cells (NK), B cell, CD4^+^, and CD8^+^ T cells, were computed based on cell-type specific DNAm sites. We included the proportions of PBL subtypes as covariates to assess the association between DNAm and smoking.

We used linear regression when identifying smoking-related DNAm sites and conducting replication analyses in the GEO datasets, as they did not contain chip/batch information. Information on BMI was not available, so associations were adjusted only for age.

We removed one outlier from the twin discovery cohort with an extreme value for BMI based on a threshold of three standard deviations from the mean, leaving 139 study participants for the chromosome-wide epigenomic analysis. No outliers were removed from the second twin cohort or the GEO data, leaving 78 twins, 464 (327 males), and 333 (95 males) individuals in the three datasets, respectively. We stratified the two GEO datasets by sex to replicate the X chromosomal associations with cigarette smoking in males and examined the associations in females separately. In addition to the replication of two smoking-related DNAm sites in males, we examined the epigenetic associations with current smoking status in 238 females (66 current smokers) from the GEO dataset GSE42861. We did not examine the association among the 137 female participants from the dataset GSE50660 due to a small sample size of female current smokers (*N* = 7), which limited the statistical power.

For initial site discovery analyses, we modeled *β*-values as the outcome and used a false-discovery rate (FDR) of 0.05 to account for multiple testing. We also examined the significant associations using *M*-values to ensure consistency in our results. For replication analyses of significant results, we ran site-specific models in the replication cohorts. An inverse variance based meta-analysis was carried out using the “meta” package in R to combine the results from discovery and replication cohorts. For those DNAm sites significantly associated with smoking, we applied Cochran’s Q test [72] to examine the heterogeneity of the results across the discovery and replication cohorts.

To understand the genetic and environmental contributions to these two smoking-related DNAm sites, we used a structural equation modeling (SEM) method implemented in OpenMX [42] to partition the additive genetic, common environmental, and unique environmental variance in a total of 81 MZ and 27 DZ twin pairs. Additionally, we examined the differential DNAm levels between 23 MZ twin pairs discordant for current smoking status using paired *t*-test. The differential methylation between MZ twin pairs is only driven by unique environmental factors.

All statistical analyses were performed in the R statistical environment version 3.1.2 (http://www.r-project.org/). R package *nlme* was used to implement linear mixed effect model.

## Additional files

Additional file 1: Figure S1. Distribution of mean β-values of all X chromosomal sites in males (A) and females (B). **Figure S2.** Quantile-quantile plot comparing observed *p*-values to expected *p*-values of all CpG sites on the X chromosome from the epigenetic association study with current smoking status. Dashed line indicates 95 % CI for distribution of expected *p*-values. **Figure S3.** Manhattan plot of all CpG sites on the X chromosome and their association with current smoking status. The red line represents a FDR significance level of 0.05. **Figure S4.** Forest plots of the smoking-related DNAm sites in males from the discovery and three replication samples using M-value. A: cg07764473 (*BCOR*). B: cg21380860 (*TSC22D3*). (PDF 703 kb)
