# Supplementary material for: X chromosome-wide analysis identifies DNA methylation sites influenced by cigarette smoking
Source: Clin Epigenetics. 2016 Feb 24;8:20. doi: 10.1186/s13148-016-0189-2 (PMC4765206; doi:10.1186/s13148-016-0189-2)

Figure S1. Distribution of β-values of all X chromosomal sites in males (A) and females (B).

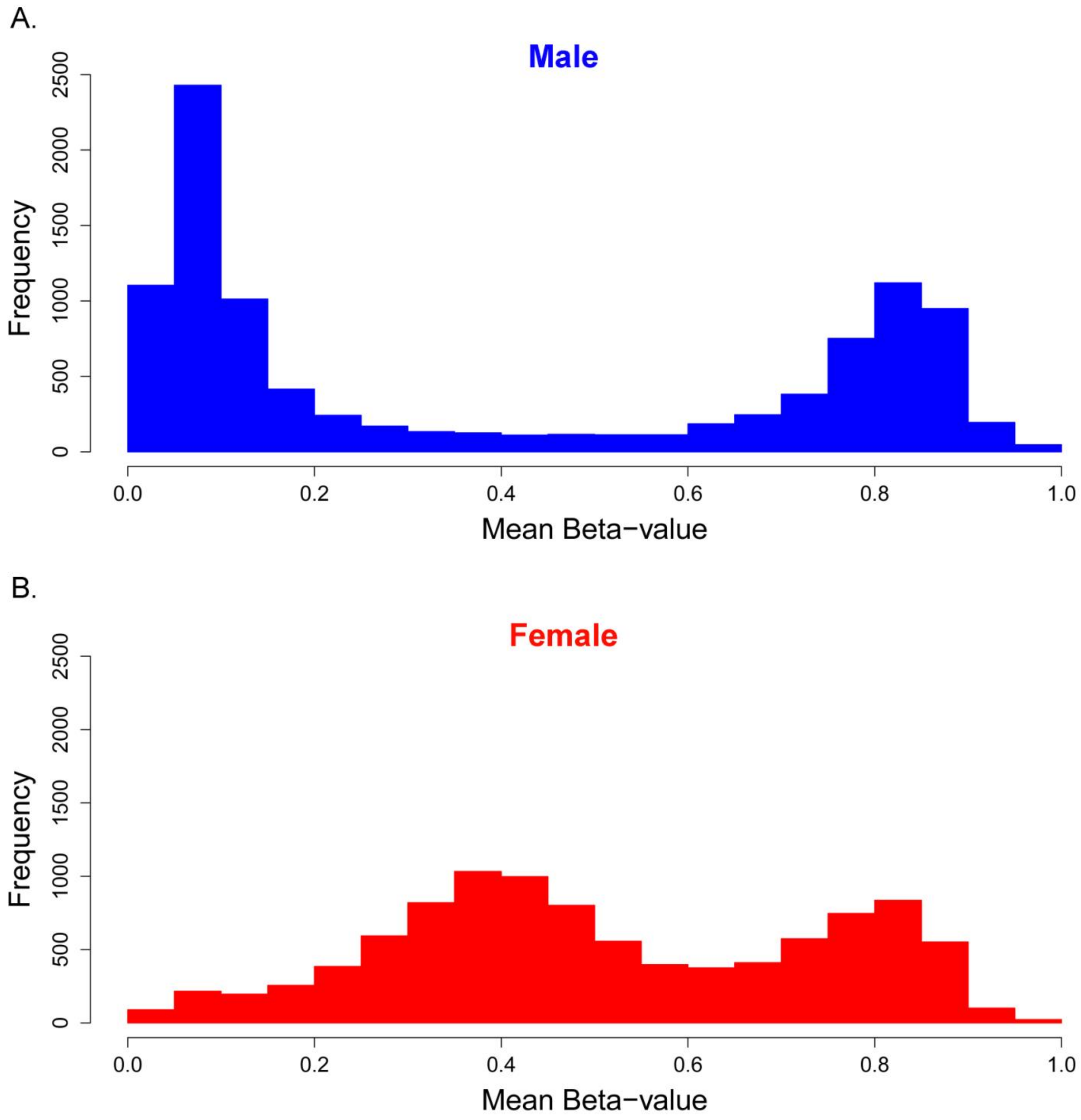

Figure S2. Quantile-quantile plot comparing observed p-values to expected p-values of all CpG sites on the X-chromosome from the epigenetic association study with current smoking status. Dashed line indicates 95% CI for distribution of expected p-values.

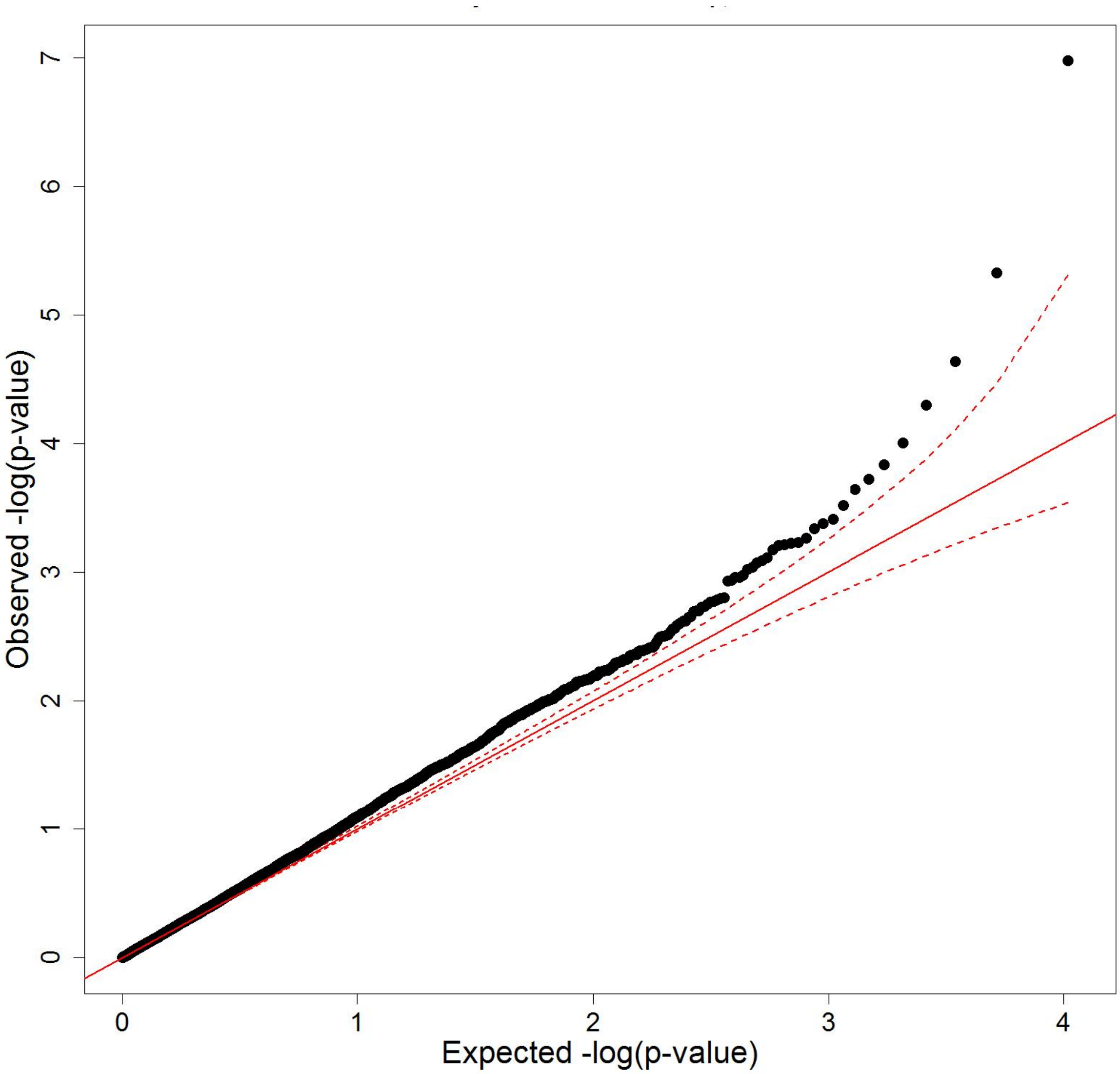

Figure S3. Manhattan plot of all CpG sites on the X-chromosome and their association with current smoking status. The red line represents a FDR significance level of 0.05.

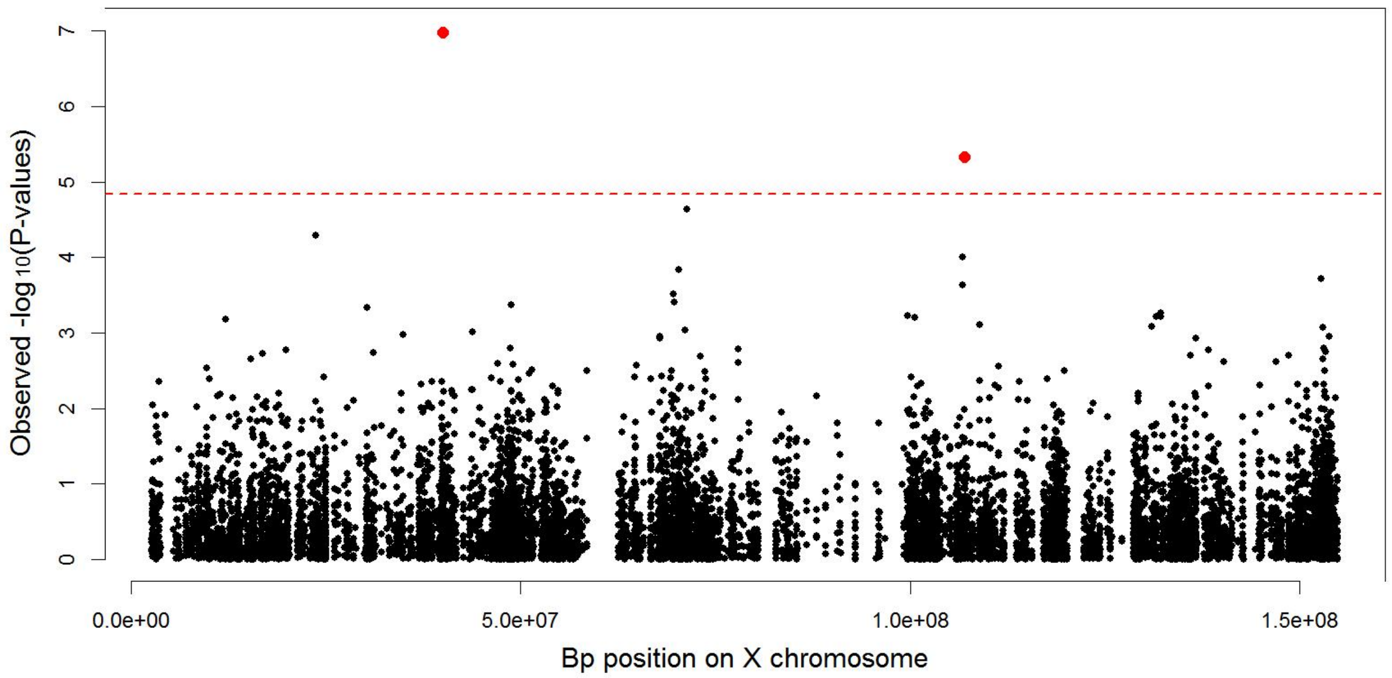

Figure S4. Forest plots of the smoking-related DNAm sites in males from the discovery and three replication samples using M-value. A: cg07764473 (BCOR). B: cg21380860 (TSC22D3).

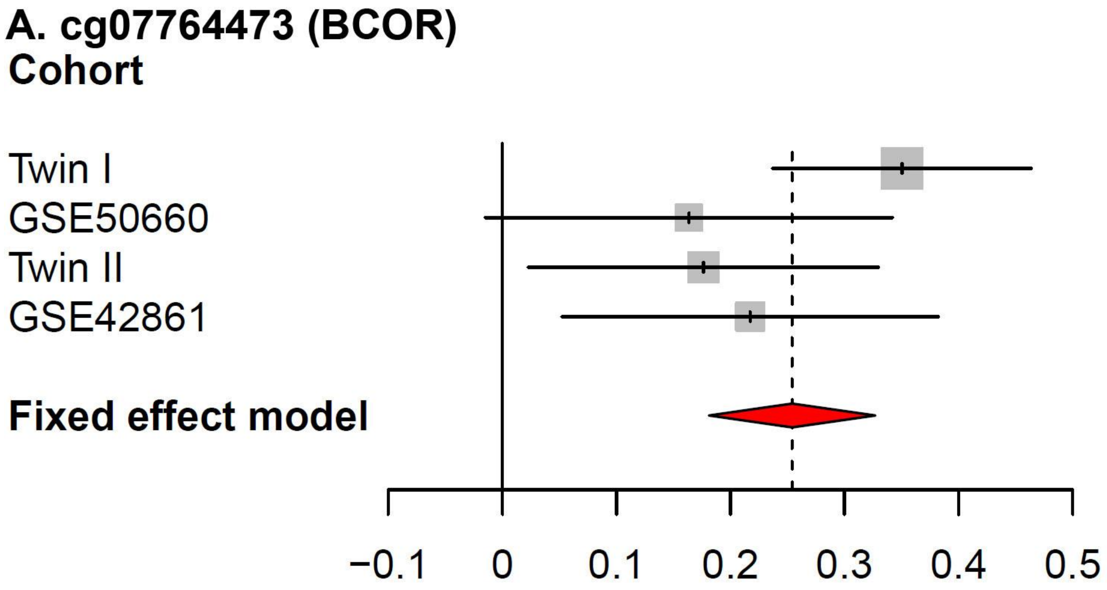

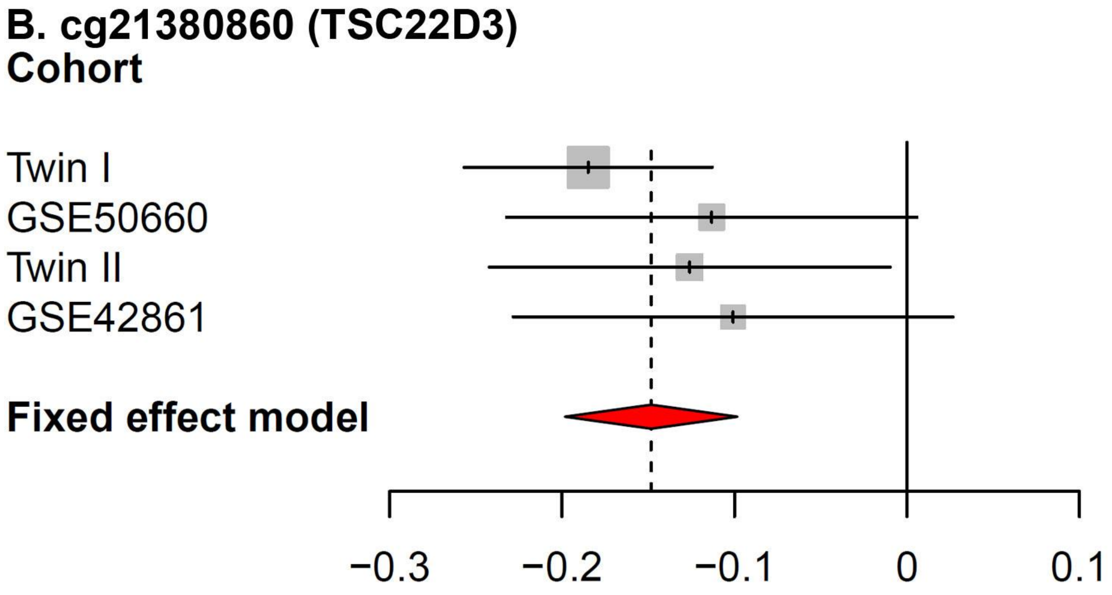

Supplement: Additional file 1: Figure S1. — Distribution of mean β-values of all X chromosomal sites in males (A) and females (B). Figure S2. Quantile-quantile plot comparing observed p-values to expected p-values of all CpG sites on the X chromosome from the epigenetic association study with current smoking status. Dashed line indicates 95 % CI for distribution of expected p-values. Figure S3. Manhattan plot of all CpG sites on the X chromosome and their association with current smoking status. The red line represents a FDR significance level of 0.05. Figure S4. Forest plots of the smoking-related DNAm sites in males from the discovery and three replication samples using M-value. A: cg07764473 (BCOR). B: cg21380860 (TSC22D3). (PDF 703 kb) [file 13148_2016_189_MOESM1_ESM.pdf]
